# Level-specific associations of urinary antimony with cognitive function in US older adults from the National Health and Nutrition Examination Survey 2011–2014

**DOI:** 10.1186/s12877-022-03351-6

**Published:** 2022-08-12

**Authors:** Xiangdong Wang, Rui Wang, Zeyao Zhang, Chao Luo, Zixuan Zhao, Junpu Ruan, Rongrong Huang, Hongbing Zhang, Qiyun Wu, Shali Yu, Juan Tang, Xinyuan Zhao

**Affiliations:** 1 Department of Occupational Medicine and Environmental Toxicology, Nantong Key Laboratory of Environmental Toxicology, School of Public Health, Nantong University, Nantong, 226019 China; 2 Department of Pharmacy, Affiliated Hospital of Nantong University, Nantong, China; 3 Jiangsu Provincial Center for Disease Control and Prevention, Nanjing, 210009 China

**Keywords:** Urinary antimony, Cognitive function, NHANES, Older

## Abstract

**Background:**

We have looked at antimony (Sb) as a new neurotoxin which causes neuronal apoptosis in animal studies. At the population level, however, there is no direct evidence for a relationship between Sb exposure and cognitive performance.

**Method:**

The study comprehensively assessed the correlation between urinary antimony levels and cognitive test scores in 631 creatinine-corrected older persons using data from the National Health and Nutrition Examination Survey (NHANES) from 2011 to 2014.

**Results:**

Using logistic regression, the study looked at the prevalence of cognitive impairment at different levels of urine antimony concentrations and found that, after controlling for covariates, higher doses of urinary antimony were positively associated with cognitive function compared to controls, odds ratio (ORs) with 95% confidence interval (CI) were 0.409 (0.185–0.906) and 0.402 (0.186–0.871) respectively. Restricted cubic spline curves showed a non-linear and dose-specific correlation between urinary antimony and cognitive performance, with lower doses associated with better cognitive performance, while higher doses may be associated with cognitive impairment.

**Conclusions:**

Our data provide evidence for a correlation between Sb and cognitive function at the population level, although the specific mechanisms need to be investigated further.

**Supplementary Information:**

The online version contains supplementary material available at 10.1186/s12877-022-03351-6.

## Introduction

The prevalence of cognitive impairment continues to climb as the world’s population ages. Dementia in all forms, ranging from mild cognitive impairment (MCI) to Alzheimer’s disease (AD), is rapidly becoming a serious global public health issue [1]. Dementia is the most common brain condition, characterized by severe cognitive impairment and may be associated with inflammatory responses and oxidative stress [2]. MCI is a transitional stage between the expected cognitive decline of normal aging and the expected cognitive decline of dementia [3]. Within three years of diagnosis, the majority of persons with MCI develop dementia, including Alzheimer’s disease, Lewy body disease and frontotemporal lobar dementia [4–6]. AD, as the commonest cause of dementia, is a catastrophic neurodegenerative disease that afflicts approximately 40 million people worldwide, even predicted to increase to 50 million people until 2030 [7]. Until now, there are still no effective treatments to reverse or slow the AD progression [8]. It is inevitable that prevalent and a worsening healthcare burden is increased [9].

Not only pathology, but also the causative factors of MCI, are extremely complex. There is no doubt that aging is a widely-accepted factor in the pathogenesis of MCI, because MCI mainly occurs in the older. Moreover, poor lifestyle is critical to the onset of cognitive impairment. Among which, smoking and drinking are one of the two most important factors. The effect of smoking on cognitive impairment may be partially due to the neurotoxicity of nicotine and to the contribution of smoking to many chronic diseases [10]. Alcohol intake within the recommended limits was not significantly associated with risk of dementia, but chronic high-dose drinking may be associated with cognitive impairment [11]. Long-term metal exposure has been linked to brain dysfunction, cognitive decline in adults, and negative effects on attention, executive function, mental flexibility, and cognitive efficiency, according to recent research [12]. This may be related to trace metal dyshomeostasis. With the aggressive pace of human activity and the release of excess metals into the environment, long-term exposure of the public to a wide range of heavy metals is becoming increasingly common [13]. The presence of heavy metals such as aluminium, arsenic, copper, lead, mercury and zinc may enhance levels of oxidative stress in the brain, leading to neuronal toxicity [14]. Arsenic, lead, cadmium and tungsten were significantly and negatively correlated with cognitive test scores [15]. In addition, excessive manganese exposure leads to manganese accumulation in the cerebral cortex and hippocampus, potentially impairing cognitive function [16, 17]. Population studies have also confirmed that manganese exposure is associated with a significant reduction in cognitive function in older adults [18].

Antimony (Sb), a metalloid with atomic number 51, belongs to group 15 of the periodic table, existing naturally in rocks, water and soils [19]. Antimony Trioxide is reasonably considered to be a human carcinogen according to the latest Carcinogen report from United States Department of Health and Human Services (HHS) [20]. In general, respiratory and cardiovascular systems are thought to be main targets after Sb exposure [21]. Following investigation of Tanu et al., several studies from our group suggest that Sb may also be neurotoxic. We report in detail that Sb stimulates apoptosis in mouse neurons and PC12 cells through reactive oxygen species-dependent autophagy activation and beta-catenin downregulation [22, 23]. Moreover, we also found that Sb increased AD-like pathological changes in mice brain [24]. As another neurotoxicity-associated changes in neuronal cell behavior, astrocytes activation was also stimulated after Sb treatment via nuclear factor-kB and CREB phosphorylation [25, 26]. Reactive astrocytes were reported to produce pro-inflammatory factors, therefore inducing synaptic disturbances and neuritic dystrophy in several AD animal models [27]. Taken together, our experimental results of cells and animals can support AD risk of antimony. However, the population evidence for cognitive impairment risk of antimony remains limited.

Our objective is to provide population evidence for the relationship between antimony and cognitive performance and to provide some new understanding for the Sb-mediated neurotoxicity. with the help of epidemiological data from United States National Health and Nutrition Examination Survey (NHANES), we conducted an investigation on the association between urinary antimony levels in the representative U.S. older population and cognitive ability.

## Methods

### Study population

**Fig. 1:**
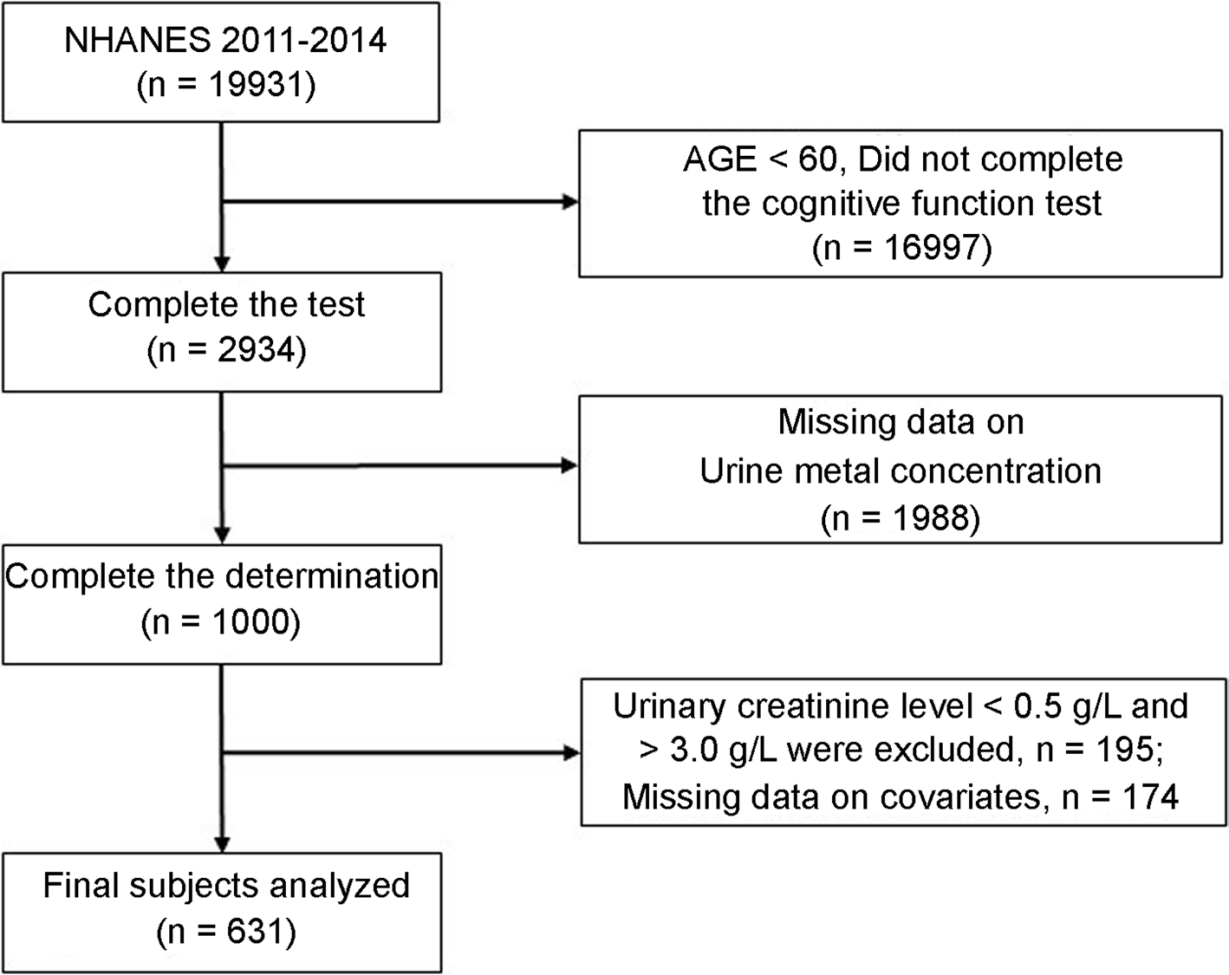
Flow chart of the selection of eligible subjects

The National Center for Health Statistics (NCHS), part of the National Centers for Disease Control and Prevention, conducts the National Health and Nutrition Examination Survey (NHANES) to collect data on the health and nutrition status of adults and children in the United States. The project uses a multi-stage sampling approach to obtain a nationally representative sample of approximately 5000 individuals per year, distributed across the country in counties, with 15 counties randomly visited each year [28]. Two cycles were combined, 2011–2012 and 2013–2014, since these two cycles for the older population were used to conduct a cognitive function score survey. These two cycles had a total of 19,931 participants at the start. People younger than 60 years old, as well as those who failed to complete the cognitive function exam and submit urine antimony data, were removed from the study (*n* = 18,985). Urine samples having creatinine concentrations of more than 3 g/L or less than 0.5 g/L were not utilized for creatinine correction of urine antimony concentration. Among these subjects, Urinary creatinine concentration meets the inclusion criteria in 805 participants, and 631 participants had data for all covariates (Fig. 1).

### Cognitive performance test

NHANES performed a series of assessments for cognitive performance among participants aged 60 years or older by the way of the Consortium to Establish a Registry for Alzheimer’s Disease (CERAD) Word List Learning Test, the CERAD Word List Recall Test, the Animal Fluency test (AFT) and the Digit Symbol Substitution Test (DSST) at Mobile Examination Center (MEC) [29, 30]. While the results of these tests cannot replace the diagnosis based on clinical examinations, they have been used to investigate the relationship between cognitive function and a variety of risk factors [15, 31].

The word learning and recall module in CERAD is designed to test cognitive functioning and to assess immediate and delayed learning of new verbal information in the memory sub-domain [32]. Participants in the learning experiment were asked to read aloud 10 unrelated words. Following the presentation of the terms, the participants were asked to recall as many as possible. Each of the three learning trials had a different order of 10 words, with a maximum score of 10 points for each trial. Delayed testing is typically done after the completion of animal’s fluency and DSST tests. The number of correct responses in each trial determines the trial’s score, which ranges from 0 to 10. CERAD’s overall score is based on the results of three immediate trials and one delayed trial (CERAD-Total). The Animal Fluency test looks at categorical verbal fluency, which is a part of executive function. Its results have been shown to distinguish between persons who have normal cognitive functioning and those who have mild cognitive impairment or more severe forms of cognitive impairment, such as Alzheimer’s disease [33]. Participants were given a minute to name as many animals as they could in under a minute, with each named animal receiving one point. The Digit Symbol Substitution test (DSST), a performance module from the Wechsler Adult Intelligence Scale (WAIS III), which requires the integrity of executive function, processing speed, attention, spatial perception, and visual scanning [34]. The subjects were required to do this assignment on a sheet of paper. There are 9 numbers, some symbols, and a legend. Each number has a symbol associated with it. Within 120 seconds, the examinee must match as many symbols and numbers as feasible according to the coding table. The total number of right matches determines the score, which ranges from 0 to 133.

For CERAD, AFT, and DSST results, there is currently no gold standard cut-off point for detecting impaired cognitive performance. To highlight different forms of cognitive performance, we used the lowest quartile of each test score in the study group as a cut-off, which is consistent with earlier published literature [35].

### Measurement of urinary antimony levels

Professionals took urine samples at the testing location. They were immediately kept in an environment below 4 °C after collection, then transferred to the CDC Environmental Health Center in Atlanta, Georgia, USA, where they were maintained in an environment below minus 70 degrees and analyzed within three weeks of collection. Urinary antimony concentrations were determined by inductively coupled plasma mass spectrometry with a lower limit of detection (LLOD, μg/L) of 0.022 μg/L [36].

### Covariates

According to the previously related publications, the following covariates are included in this study that are thought to be related to cognitive function: age group (60 to <70 years, 70 to <80 years, and ≥ 80 years), sex (male and female), race/ethnicity (Mexican American, non-Hispanic white, other Hispanic, non-Hispanic black and other race), educational level (below high school, high school, above high school), marital status (married/living with partner and widowed/divorced/separated/never married), poverty–income ratio (under poverty level: ≤1, above poverty level: >1) [37], body mass index (BMI) (normal: <25 kg/m^2^, overweight: 25 to <30 kg/m^2^, obese: ≥30 kg/m^2^), smoking status (current, ever, never), drinking status (<12drinks/year and ≥ 12drinks/year). The categories for hypertension, diabetes, chronic bronchitis, renal failure [38], coronary heart disease, and arthritis are based on whether or not they have ever been informed by a doctor or other health expert. Subjects were asked by trained professionals, “Have you experienced confusion or memory loss in the past 12 months? (difficulty in thinking: yes or no)”.

### Statistical analysis

To account for the complicated sample design and ensure that the estimates are nationally representative, NHANES uses a stratified, multi-level probability clustering procedure as a sampling approach. All statistical analysis is adjusted for the survey design and weighting factors. The 2-year weights (wtsa2yr) were chosen for the urinary metals testing subsamples in NHANES 11–12 and 13–14 according to the NHANES analytical criteria. When the two survey cycles were joined, the ultimate weight (1/2 × wtsa2yr for NHANES 11–14) was adjusted. Considering the effect of variation in urine concentration on the concentration of the measured substance in the urine, we separated the creatinine-corrected urine antimony data into four equal portions based on the quartiles and transformed them to ordinal categorical variables. Based on cut-offs of <13 for AF, <32 for DSST, <20 for CERAD-Total were used to distinguish potential cognitive impairments from healthy cognitive functions in research population. The percentages of categorical variables were compared among the groups using Chi-square testing. To describe the antimony concentration in urine, we employ the geometric mean, confidence interval, and median (interquartile range). The dose-response association between urine antimony concentrations and cognitive performance was investigated using logistic regression and restricted cubic spline. Based on previous research and theoretical considerations, we included previously reported factors that may affect outcomes (age, sex, BMI, education, race, poverty-to-income ratio, marital status, drink, smoke, hypertension and diabetes), and chi-square tests Factors found to be statistically different in all three cognitive tests (difficulty in thinking) were used as adjustment variables for multivariate logistic regression. In binary logistic regression, different potential confounders were adjusted in Model 1 (did not adjust any confounders), Model 2 (Adjust to the characteristics of the population, age, sex, BMI, education, race, poverty-to-income ratio, marital status, drink and smoke) and Model 3 (Model 3 was adjusted for all variables in Model 2 as well as other confounders including difficulty in thinking, hypertension and diabetes). ORs and 95% CIs were calculated for the prevalence of cognitive impairment in quartile 2 (Q2), quartile 3 (Q3) and quartile 4 (Q4) respondents compared to quartile 1 (Q1) for urinary antimony levels.

All statistical analyses were conducted by R version 4.1.1, P values <0.05 were considered statistically significant.

## Results

This study comprised a total of 631 older persons, all of whom were 60 years or older (Table 1). The mean age of participants was 69.5 (± 6.6) years (range: 60–80), and women represented 45.2% of the overall sample. Of all subjects included in this study, 400 (63.39%) cases with hypertension, 148 (23.45%) with diabetes, 44 (6.97%) with renal failure, 324 (51.35%) with arthritis, 54 (8.56%) with coronary heart disease, 49 (7.77%) with chronic bronchitis and 101 (16.01%) with difficulty in thinking were identified. Among the three different measures of cognitive function, there are considerable disparities in age distribution and educational level between people with cognitive impairment and those with normal cognitive performance. However, these differences did not exist between BMI and chronic bronchitis. Furthermore, participants with cognitive impairment were more likely to have a lower educational level, and inferior cognitive performance was more common in males and the low-income group. This table also reveals the geometric mean and quartiles of creatinine-adjusted urinary antimony content. The geometric mean of the U-Sb level was 0.0469 μg/g creatinine with a 95% confidence interval of 0.0445 to 0.0494 μg/g creatinine. The median is 0.0443 μg/g creatinine, and the upper and lower quartiles are 0.03133 and 0.06524 μg/g creatinine, respectively. In addition, we calculated mean score for each cognitive function score by creatinine-adjusted quartiles of urinary antimony levels (Table 2).

**Table 1:** Characteristics of the study population, National Health and Nutrition Examination Survey (NHANES) 2011–2014 (*N* = 631)

catalogues	CERAD Test			Animal Fluency Test			Digit Symbol Test		
	Normal	Low	*p* value	Normal	Low	*p* value	Normal	Low	*p* value
	Cognitive	Cognitive		Cognitive	Cognitive		Cognitive	Cognitive	
	Performance	Performance		Performance	Performance		Performance	Performance	
**Number of subjects (%)**	502	129		477	154		483	148	
**Age (%)**^a^			<0.01			<0.01			0.031
60–70 years	275 (54.78)	57 (44.19)		267 (55.97)	65 (42.21)		268 (55.49)	64 (43.24)	
70–80 years	169 (33.67)	38 (39.46)		152 (31.87)	55 (35.71)		150 (31.06)	57 (38.51)	
≥80 years	58 (11.55)	34 (26.35)		58 (12.16)	34 (22.08)		65 (13.46)	27 (18.24)	
**Sex (%)**^a^			<0.01			0.992			0.0198
Male	261 (51.99)	85 (65.89)		261 (54.72)	85 (55.19)		252 (52.17)	94 (63.51)	
Female	241 (48.01)	44 (34.11)		216 (45.28)	69 (44.81)		231 (47.83)	54 (36.49)	
**Race (%)**^a^			0.106			<0.01			<0.01
Mexican American	30 (5.98)	14 (10.85)		29 (6.08)	15 (9.74)		19 (3.93)	25 (16.89)	
Other Hispanic	47 (9.36)	19 (14.73)		53 (11.11)	13 (8.44)		39 (8.07)	27 (18.24)	
Non-Hispanic White	242 (48.21)	55 (42.64)		241 (50.52)	56 (36.36)		252 (52.17)	45 (30.41)	
Non-Hispanic Black	138 (27.49)	31 (24.03)		114 (23.90)	55 (35.71)		123 (25.47)	46 (31.08)	
Other race	45 (8.96)	10 (7.75)		40 (8.39)	15 (9.74)		50 (10.35)	5 (3.38)	
**Educational level (%)**^a^			<0.01			<0.01			<0.01
Below high school	109 (21.71)	53 (41.09)		107 (22.43)	55 (35.71)		78 (16.15)	84 (56.76)	
High school	113 (22.51)	27 (20.93)		93 (19.50)	47 (30.52)		110 (22.77)	30 (20.27)	
Above high school	280 (55.78)	49 (37.98)		277 (58.07)	52 (33.77)		295 (61.08)	34 (22.97)	
**Marital status (%)**^a^			0.99			0.218			0.035
Married/living with partner	293 (58.37)	76 (58.91)		286 (59.96)	83 (53.90)		294 (60.87)	75 (50.68)	
Widowed/divorced/separated/never married	209 (41.63)	53 (41.09)		191 (40.04)	71 (46.10)		189 (39.13)	73 (49.32)	
**Poverty–income ratio (%)**^a^			0.268			<0.01			<0.01
≤0.99	89 (17.73)	29 (22.48)		76 (15.93)	42 (27.27)		67 (13.87)	51 (34.46)	
≥1	413 (82.27)	100 (77.52)		401 (84.07)	112 (72.73)		416 (86.13)	97 (65.54)	
**Body mass index (%)**^a^			0.258			0.73			0.538
< 25 kg/m^2^	117 (23.31)	32 (24.81)		109 (22.85)	40 (25.97)		119 (24.64)	30 (20.27)	
25–30 kg/m^2^	179 (35.66)	54 (41.86)		178 (37.32)	55 (35.71)		175 (36.23)	58 (39.19)	
≥ 30 kg/m^2^	206 (41.04)	43 (33.33)		190 (39.83)	59 (38.31)		189 (39.13)	60 (40.54)	
**Smoking status (%)**^a^			0.559			0.376			0.011
Never	237 (47.21)	64 (49.61)		224 (46.96)	77 (50.00)		235 (48.65)	66 (44.59)	
Former	210 (41.83)	55 (42.64)		207 (43.40)	58 (37.66)		208 (43.06)	57 (38.51)	
Current	55 (10.96)	10 (7.75)		46 (9.64)	19 (12.34)		40 (8.28)	25 (16.89)	
**drinking status (%)**^a^									
<12drinks/year	349 (70.51)	81 (65.32)	0.312	336 (71.79)	94 (62.25)	0.034	335 (70.38)	95 (66.43)	0.427
≥12drinks/year	146 (29.49)	43 (34.68)		132 (28.21)	57 (37.75)		141 (29.62)	48 (33.57)	
**Hypertension (%)**^a^	316 (62.94)	84 (65.12)	0.724	283 (59.33)	117 (75.97)	<0.01	303 (62.73)	97 (65.54)	0.601
**Diabetes (%)**^a^	114 (22.71)	34 (26.37)	0.45	96 (20.13)	52 (33.77)	<0.01	100 (20.7)	48 (32.43)	<0.01
**Renal failure (%)**^a^	33 (6.57)	11 (8.53)	0.56	30 (6.29)	14 (9.09)	0.315	24 (4.97)	20 (13.51)	<0.01
**difficulty in thinking (%)**^a^	65 (12.95)	36 (27.91)	<0.01	65 (13.63)	36 (23.38)	<0.01	68 (14.08)	33 (22.3)	0.024
**Arthritis (%)**^a^	265 (52.79)	59 (45.74)	0.183	245 (51.36)	79 (51.29)	0.99	257 (53.21)	67 (45.27)	0.11
**Coronary heart disease (%)**^a^	34 (6.77)	20 (15.5)	<0.01	34 (7.13)	20 (12.99)	0.036	38 (7.87)	16 (10.81)	0.34
**Chronic bronchitis (%)**^a^	40 (7.97)	9 (6.98)	0.849	42 (8.81)	7 (4.55)	0.123	39 (8.07)	10 (6.76)	0.727
**U-Sb concentration**	0.0469 (0.0445, 0.0494) ^b^				0.04430 (0.03133, 0.06524) ^c^				

^a^ Chi-square test was used to compare the percentage between participants low and normal cognitive performance

^b^ G-Mean (95%)

^c^ Median (25th, 75thpercentiles)

**Table 2:** Mean score for each cognitive function score by quartile of creatinine-adjusted urinary antimony levels

Catalogues	CERAD Test ^b^	Animal Fluency Test ^b^	Digit Symbol Test ^b^	Sb (μg/g creatinine) ^c^
All subjects	24.84 (6.42)	16.71 (5.59)	45.12 (17.16)	0.04408
Q1 (<0.03127) ^a^	24.58 (6.72)	16.59 (5.69)	44.97 (17.59)	0.02343
Q2 (> = 0.03127 & <0.04408) ^a^	25.06 (6.52)	16.83 (5.32)	45.48 (17.35)	0.03676
Q3 (> = 0.04408 & <0.06524) ^a^	25.04 (5.94)	16.98 (5.57)	45.30 (17.58)	0.05179
Q4 (> = 0.06524) ^a^	24.69 (6.53)	16.42 (5.78)	44.74 (16.22)	0.09318

^a^ Quartiles of creatinine-adjusted urinary antimony levels

^b^ Mean (SD)

^c^ Medians for each quartile

**Fig. 2:**
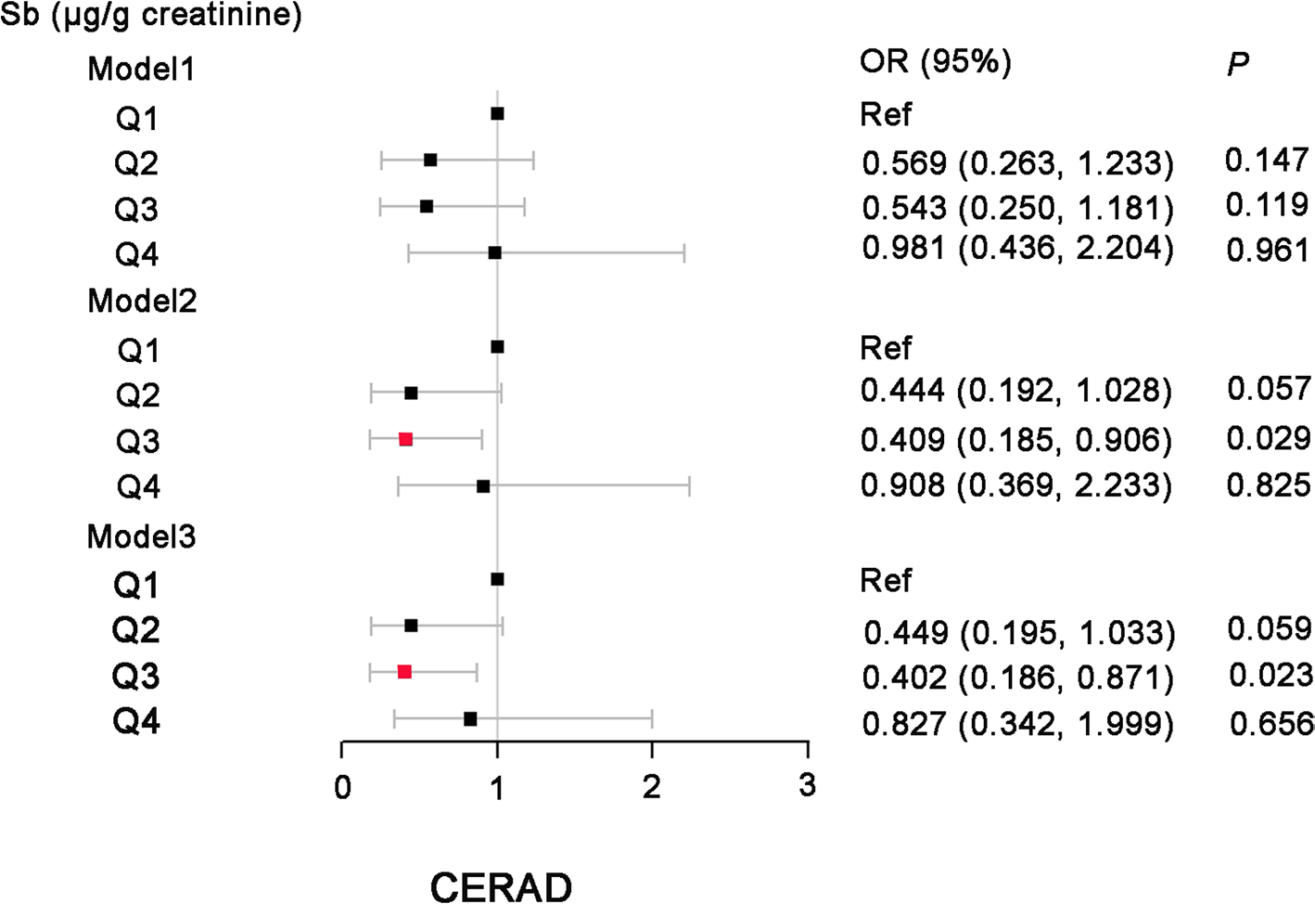
Weighted odds ratios (95% confidence intervals) of cognitive impairment by quartiles of urine antimony level. The black squares and horizontal line represent for the OR and 95% confidence interval, the red squares represent statistically significant differences

In general, the results of the three models of multiple logistic regression are similar (Fig. 2, Fig. S1). Compared to the lowest quartile of U-Sb, Model 1 calculates a crude OR with 95% CI for CERAD-Total score, AFT and DSST results. However, none of the analyses were significant in this model (all P-values >0.05). After adjusting for covariates such as age and sex (Model 2), the OR (95% CIs) of cognitive impairment for Q3 vs. Q1 of urine antimony concentrations was 0.409 (0.185–0.906) for CERAD-Total score. In multivariable logistic analysis (Model 3), U-Sb was still associated with cognitive performance, compared to the lowest quartile of U-Sb, OR with 95% CIs were 0.402 (0.186–0.871).

**Fig. 3:**
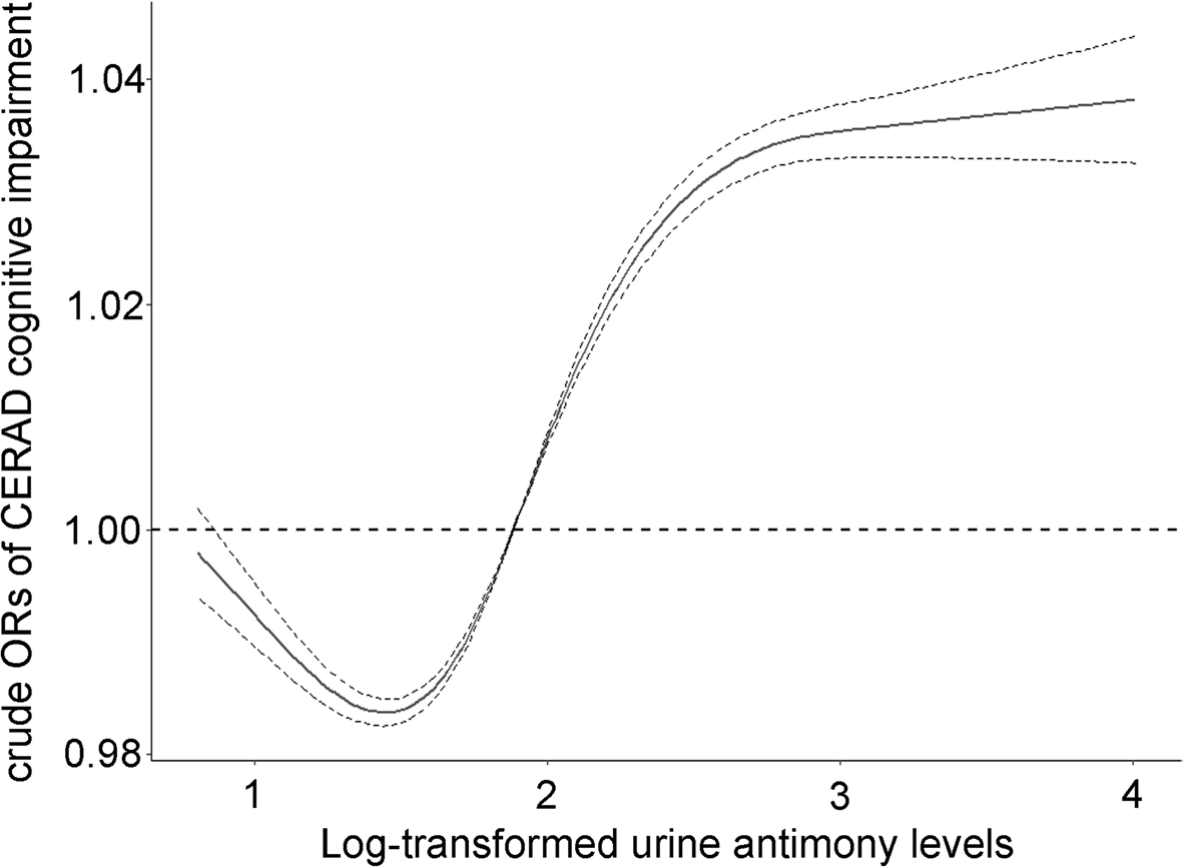
The continuous relationship between urinary antimony levels and CERAD cognitive test scores based on a restricted cubic spline regression model. The solid lines represent the ORs, and dashed lines represent the 95% CIs

Considering that urinary antimony in the population has a significant skewed distribution, and to reduce the effect of extreme values on model estimates, we logarithmically urinary antimony levels and weighted them according to the final weights. Log-transformed urinary antimony levels are nonlinearly associated with cognitive performance, we observed that below a urinary antimony level of 0.0443 μg/g creatinine, the odds of cognitive impairment assessed by CERAD were significantly decreased. And when the urinary antimony concentration reached or exceeded approximately 0.0297 μg/g creatinine, the OR value increased significantly (Fig. 3).

## Discussion

The associations were analyzed between urine antimony levels and three markers of cognitive impairment in this study, which integrated two datasets (NHANES 2011–2012, NHANES 2013–2014) and included 631 US people aged 60 or older. Our findings revealed that urine antimony concentrations within a particular range are connected with cognitive performance in the older. After controlling for confounders such as age and gender, the results of our logistic regression model, based on CERAD-Total scores, showed that the prevalence of cognitive impairment was lower in subjects in the U-Sb third level group than in those in the U-Sb lowest quartile group. In addition, the results of the weighted restricted cubic spline curves indicated that urinary antimony concentrations within a certain range were positively correlated with cognitive function in the older population, with higher concentrations of U-Sb above a certain level being linked to a greater risk of cognitive impairment. Briefly, urinary antimony levels were significantly associated with the memory subdomain as assessed by CERAD but not with DSST and AFT, the discrepancy across the tests might be due to the fact that the cognitive domains addressed by each test differ. The word learning and recall module in CERAD is designed to test cognitive function and assess the ability to learn both immediate and delayed [32], the Animal Fluency test looks at categorical verbal fluency [33], and the Digital Symbol Substitution Test (DSST) [34], which places high demands on executive function, processing speed, and attention. The brain has multiple functional regions, and the difference in results may be due to the different effects of antimony on different brain regions.

The results of the multiple logistic regression models showed that different models gave different results. The reason for this outcome may be the inclusion of covariates in the model. Covariates are variables that are linearly related to the dependent variable and are controlled for by statistical techniques when exploring the relationship between the independent and dependent variables. Variables such as age and gender are important factors influencing cognitive performance and their inclusion as covariates in a multiple logistic regression model controls for the effect of confounding factors on the relationship between antimony and cognition. This may account for the lack of association in the crude logistic regression model (Model 1), but the emergence of meaningful outcomes in Model 2 and Model 3.

Oxidative stress has been connected to physical aging and has been proposed as a trigger for the advancement of ageing and numerous neurological illnesses, including Alzheimer’s disease. In a healthy organism, the generation and scavenging of oxygen free radicals occur in a dynamic balance. The body’s protective stress response can be triggered by low levels of reactive oxygen species. Reactive oxygen species are formed when oxidative damage exceeds the body’s antioxidant system’s scavenging capabilities, resulting in a reduction in antioxidants, causing a redox imbalance in the body and subsequent aberrant expression of nucleic acids, proteins, and other macromolecules [39–41]. Furthermore, it has the potential to affect mitochondrial activity and energy stability in an indirect manner [42], leading to abnormalities in cellular metabolism and, as a result, affecting the production and accumulation of amyloid beta (Aβ) and hyperphosphorylated Tau proteins [43]. These mechanisms are also the main theoretical hypotheses for the development of cognitive impairment and Alzheimer’s disease. Our prior study on the molecular mechanisms of antimony-related neurotoxicity suggested that antimony might increase reactive oxygen species production via oxidative stress [23]. The relatively low levels of urinary antimony in the participants of this study may have produced hormesis response in vivo, which may explain the negative correlation between urinary antimony and cognitive impairment shown in logistic regression.

The dose-response relationship for metals exhibits multiple patterns and are related to a variety of factors. Among them, lead and chromium are non-threshold toxicants with no safe dose and can exert toxic effects at very low doses [44]; Manganese is an essential trace metal required to maintain normal physiological function of neurons, but has toxic effects at low levels or in excess [45]; Cadmium induces cell proliferation at low concentrations but significantly inhibits cell growth at high concentrations [46]. However, the dose-response relationship of metallic antimony has not yet been definitively determined. Previous experimental results have shown that ROS is an important mechanism of action of antimony [47]. In order to verify the effect of antimony exposure on the nervous system, our research group has carried out a series of studies. On the one hand, we selected PC12 cells (dose at 0–100 μM) for in vitro experiments, and the results showed that the phenomenon of neuronal apoptosis was significantly increased [23]. With further studies by our group, we found that antimony enhances oxidative stress and increases reactive oxygen species production, which in turn inhibits the Akt/mTOR pathway, induces autophagy activation and lead to neuronal apoptosis [48]. On the other hand, Sb exposure (dose at 0–5 μM) induced astrocyte activation [26]. Astrocyte activation has been reported to prevent central nervous system (CNS) cell damage by protecting cells from oxidative stress [49, 50]. Thus, combined with the results of our population study, we can propose a hypothesis that there may be a level-specific associations of urinary antimony with cognitive function.

Antimony can exist in the living and occupational environment, enter the body through drinking water and skin contact, and cause serious and widespread biological toxicity [51]. In a cross-sectional study of the antimony factory population, antimony factory workers had antimony concentrations of 3.88 ± 1.10 μg/L, 27.15 ± 6.00 μg/g creatinine, and 0.10 ± 0.01 μg/g in blood, urine, and hair, respectively, which were higher than the urinary antimony levels of the subjects in this study [52]. Higher observed urine antimony levels may increase the prevalence of cognitive impairment, according to the restricted cubic spline results of this investigation. Previous animal investigations conducted by our research group demonstrated that antimony exposure at levels of 10 and 20 mg/kg significantly promoted Aβ and elevated tau phosphorylation at ser199 and ser396 in both brain regions of mice [24].

Before this study, Sasaki et al. [15]. used linear regression models to explore the association between urinary concentrations of 19 metals and metabolites and cognitive test scores in the NHANES dataset. And their results showed that urinary antimony concentrations were not significantly associated with cognitive function. Although this study used similar independent and outcome variables, there were significant differences in the selection of the study population. We used stricter inclusion criteria for creatinine and corrected urinary metal levels using urinary creatinine to exclude the effect of urine concentration or dilution on metal levels. In addition, before constructing statistical descriptions and conclusions, we weighted the original data. To ensure that estimates were representative of the civilian non-institutionalized population in the United States, samples were weighted to account for complicated survey design (including oversampling), survey non-responsiveness, and post-stratification [53]. Moreover, in addition to CERAD and DSST, we included AFT scores as outcome variables in the statistical model, which is a more comprehensive assessment of cognitive function, and selected different potential confounders such as BMI [54], renal failure [38] and PIR [55], based on previous studies. The above differences are also possible reasons for the different conclusions we have reached.

Our research has a number of strengths. The utilization of a nationally representative sample of older individuals from NHANES, which has high quality survey methods and quality control, is a substantial advantage. To properly evaluate the link between urine antimony levels and cognitive function, a wide variety of possible confounders were accounted for. Nonetheless, there are a few flaws in our research. In making statistical inferences, we converted the continuous variable of urinary antimony content into an ordered categorical variable, which may have masked certain characteristics of the variable and reduced statistical efficacy. Because of the cross-sectional nature of the study, these connections cannot be assumed to be causative, making it impossible to extrapolate the findings to the degree that urine antimony levels are causally linked to cognitive function. In addition, due to the cross-sectional nature of the NHANES data, participants U-Sb is more likely to reflect levels at the time of testing and we were unable to determine the temporal order of cognitive performance and urinary antimony levels. Furthermore, the range of cognitive test performed by the NHANES included Word List Learning Test, the Word List Recall Test, the AFT and the DSST, did not cover all domains of cognitive function, preventing further research into the relationship between urine antimony levels and other domains of cognitive function. Finally, due to various reasons (uncompleted cognitive tests, age restriction, urine antimony or missing covariate data), only 631 participants with all covariates were included in the study, as a result of the limited sample size and the fact that we cannot entirely rule out the potential of residual confounding, the findings may be skewed.

## Conclusion

With the development of research on metallic antimony, antimony is no longer just a common occupational health hazard, and we also need to be vigilant about the association of urinary antimony with cognitive function. Our findings reveal that urinary antimony levels are dose-specifically associated with cognitive performance in participants aged 60 or older in the United States, with lower doses associated with better cognitive performance, while higher doses may be associated with cognitive impairment, longitudinal studies are needed to validate the link between urine antimony levels and cognitive performance.

## Supplementary Information

**Additional file 1: Figure S1**. Weighted odds ratios (95% confidence intervals) of cognitive impairment by quartiles of urine antimony level. The black squares and horizontal line represent for the OR and 95% confidence interval, the red squares represent statistically significant differences.

## Data Availability

The datasets generated and/or analysed during the current study are available in the NHANES repository, https://www.cdc.gov/nchs/nhanes/index.htm.
