# Supplementary material for: Level-specific associations of urinary antimony with cognitive function in US older adults from the National Health and Nutrition Examination Survey 2011–2014
Source: BMC Geriatr. 2022 Aug 12;22:663. doi: 10.1186/s12877-022-03351-6 (PMC9375424; doi:10.1186/s12877-022-03351-6)
Supplement: Supplementary file 1 — Additional file 1: Figure S1. Weighted odds ratios (95% confidence intervals) of cognitive impairment by quartiles of urine antimony level. The black squares and horizontal line represent for the OR and 95% confidence interval, the red squares represent statistically significant differences. [file 12877_2022_3351_MOESM1_ESM.pdf]

Supplementary Figure 1

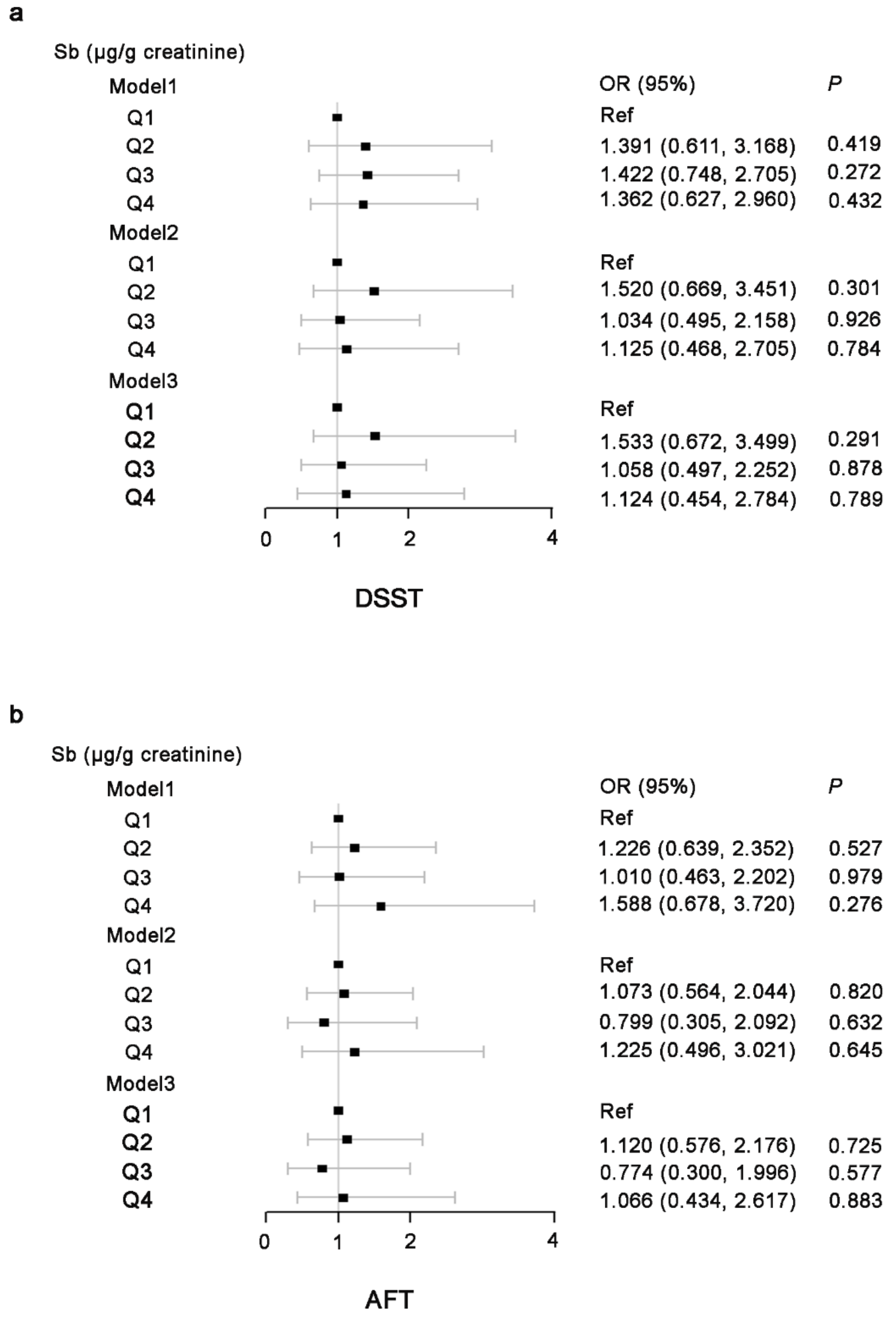

Fig S1. Weighted odds ratios (95% confidence intervals) of cognitive impairment by quartiles of urine antimony level. The black squares and horizontal line represent for the OR and 95% confidence interval, the red squares represent statistically significant differences.
